# Supplementary figures and images for: Sex-dimorphic expression of extracellular matrix genes in mouse bone marrow neutrophils
Source: PLoS One. 2023 Nov 30;18(11):e0294859. doi: 10.1371/journal.pone.0294859 (PMC10688658; doi:10.1371/journal.pone.0294859)

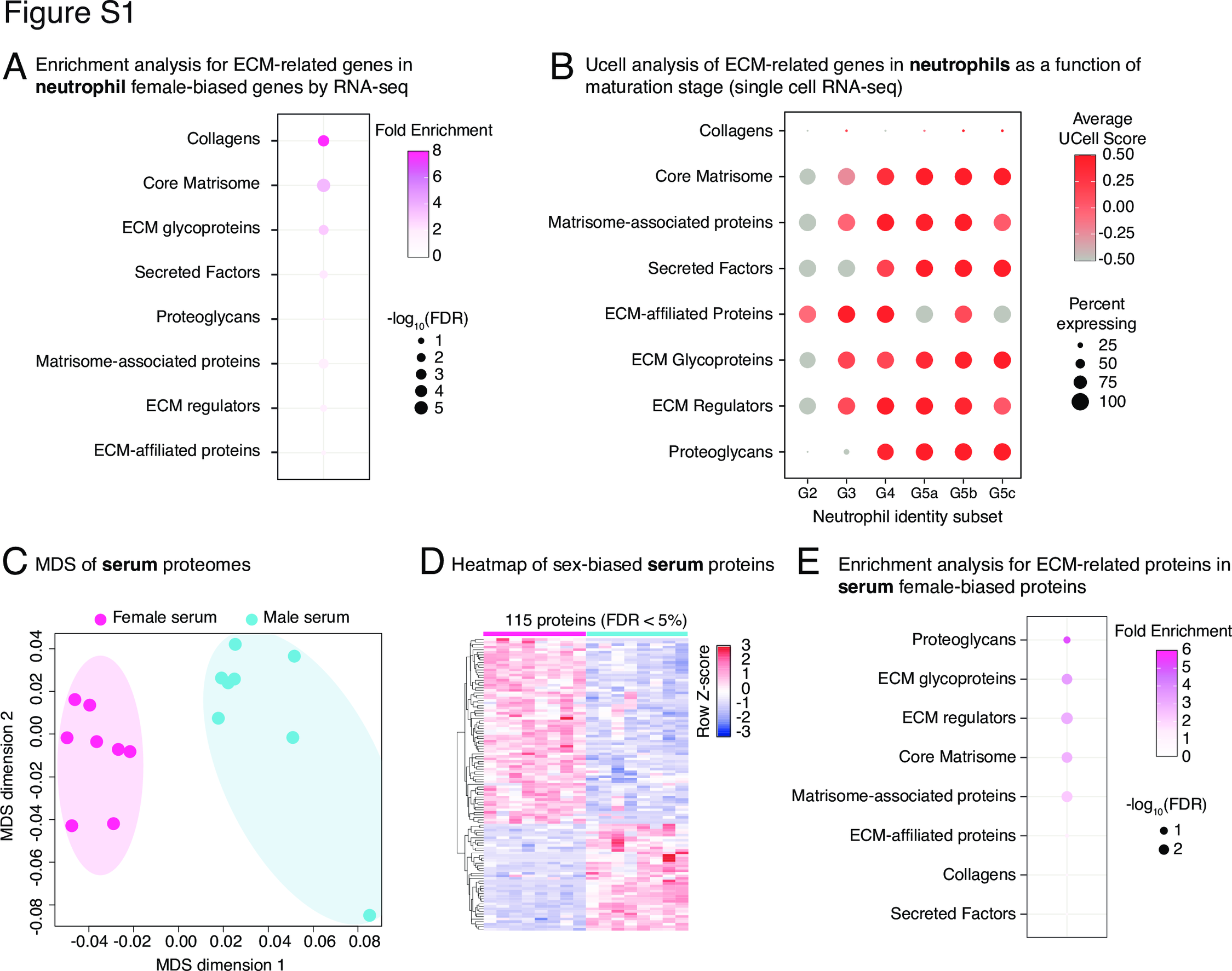

Supplement: S1 Fig — (A) Bubble plot showing Fisher’s exact test enrichment results for overlap of significantly female-biased genes in neutrophil bulk transcriptomes (DESeq2 FDR < 5%) and ECM-related genes. See full results in S2C Table. (B) Dotplot of ‘Ucell’ scores of ECM-related genesets aggregated by neutrophil maturation subset (as defined by [21]) in single-cell neutrophil RNA-seq dataset from [12]. (C) Multidimensional scaling analysis for mouse serum proteomics. MDS: Multidimensional Scaling. (D) Heatmap of significant (Limma FDR < 5%) sex-dimorphic proteins in mouse serum. Also see S2D Table. (E) Bubble plot showing Fisher’s exact test enrichment results for overlap of significantly female-biased proteins in serum proteomics (Limma FDR < 5%) and ECM-related proteins. See full results in S2E Table. (TIF) [file pone.0294859.s001.tif]

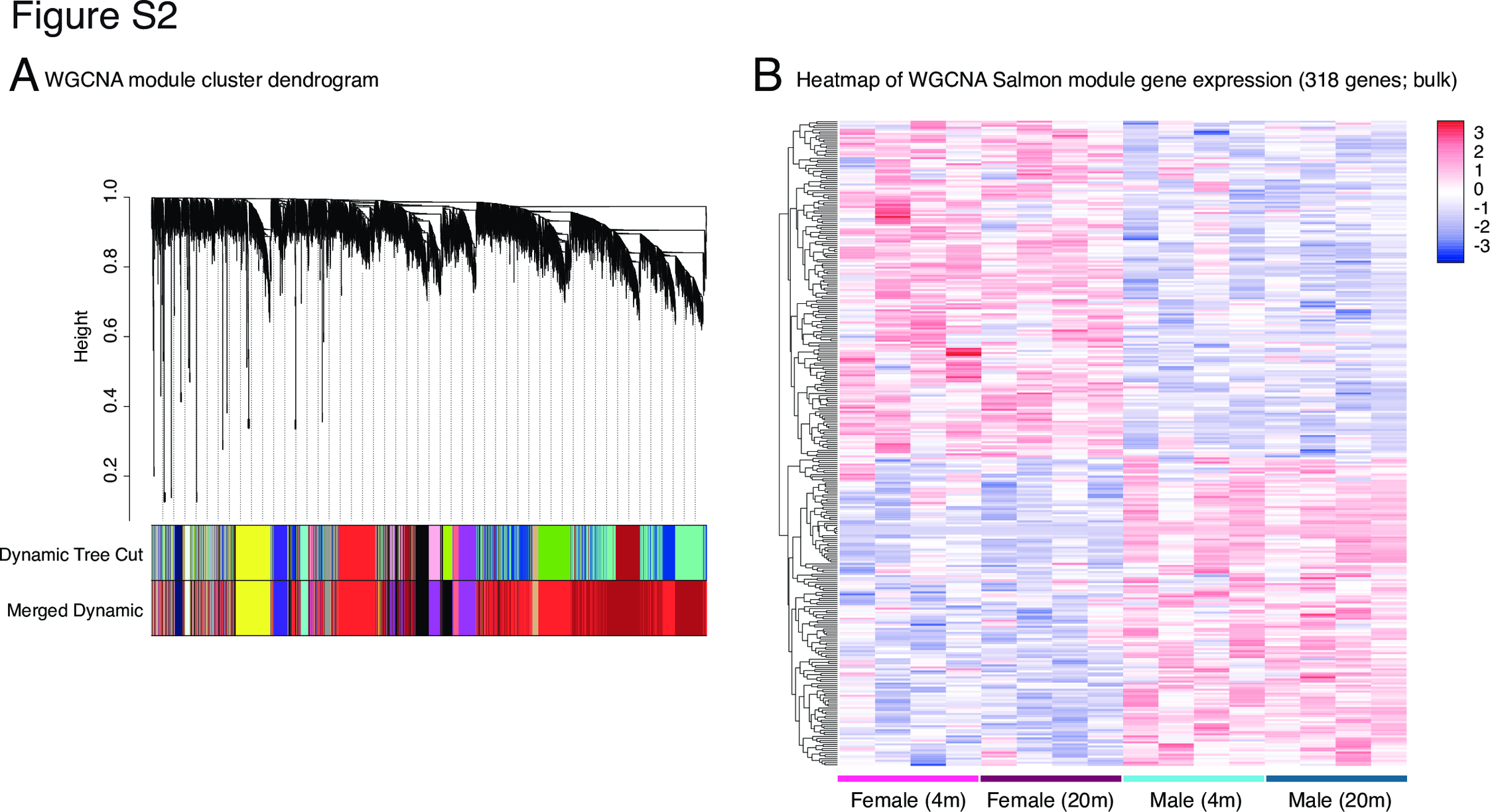

Supplement: S2 Fig — (A) WGCNA module cluster dendrogram from bulk neutrophil RNA-seq. (B) Heatmap of WGCNA Salmon module gene expression from bulk neutrophil RNA-seq. S1 Table: Curated Gene Lists for ECM-related genes. (TIF) [file pone.0294859.s002.tif]
