# Supplementary material for: Sex-dimorphic expression of extracellular matrix genes in mouse bone marrow neutrophils
Source: PLoS One. 2023 Nov 30;18(11):e0294859. doi: 10.1371/journal.pone.0294859 (PMC10688658; doi:10.1371/journal.pone.0294859)
Supplement: Attachment — Submitted filename: PONE-D-23-16855.docx [file pone.0294859.s006.docx]

In their manuscript entitled: “Sex-dimorphic expression of extracellular matrix genes in mouse bone marrow neutrophils” McGill and colleagues describe the follow up analysis stemming from their previous work, on the sex-dimorphic expression, in neutrophils, of extra-cellular matrix proteins and of the associated enzymes and other proteins.

The manuscript is generally well written, with all results available as supplementary tables, and the analyses presented mostly support the conclusions reached by the authors. However, it is unclear how the paucity of expression of the different collagen genes in single cell does affect the analysis. It is also a pity to see that there is no analysis of publicly available human datasets (Chen et al, DOI: 10.1016/j.cell.2016.10.026 or <https://maayanlab.cloud/archs4/download.html> ) to confirm that the findings made using mouse as a model system can be extended to humans and therefore being relevant for human health, as the authors conclude in their discussion.

Few minor criticisms:

1) neutrophils are the most abundant white blood cell type but not the most abundant blood cell type.
2) I would suggest avoiding the use of adjectives that are open to subjective interpretation, such as tantalising.
3) Instead of (i.e. matrisome), I would use (hereafter matrisome)
4) Figure 1, probably unintentional but I would suggest stopping the perpetuating the stereotype to assign the colours pink and blue to discriminate between females and males.
5) Lines 102/122 Fisher’s exact test is used but FDR results are shown, could the authors please explain which is correct?
6) WGCNA analysis, I am well aware that the colours are the output of the algorithm. However, to support colour blind colleagues and other readers it would be better to replace colours with numbers.
7) Figure 2C, the gene ration legend is incomplete.
